# Supplementary material for: Safety culture in French nursing homes: A randomised controlled study to evaluate the effectiveness of a risk management intervention associated with care
Source: PLoS One. 2022 Dec 1;17(12):e0277121. doi: 10.1371/journal.pone.0277121 (PMC9714758; doi:10.1371/journal.pone.0277121)
Supplement: S1 Table — (DOC) [file pone.0277121.s001.doc]

# Supplementary Material 1

Table S1: Respondent characteristics for the 2016 and 2017 surveys.

| **Professional field** | **2016** | **2017** | ***p*-value** |
| --- | --- | --- | --- |
| Paramedical | 42.9% | 46.5% | **0.038** |
| Administration/logistics/technical | 8.8% | 8.4% | 0.679 |
| Educational/ Psycho-social | 3.2% | 2.8% | 0.402 |
| Doctor | 2.9% | 1.8% | **0.001** |
| Others | 1.8% | 1.8% | 0.956 |
| Do not wish to answer/missing data | 40.4% | 39.0% | 0.477 |
| **Age bracket** | | | |
| Under 25 years old | 5.8% | 8.1% | 0.002 |
| 26 to 35 | 20.4% | 20.7% | 0.801 |
| 36 to 45 | 23.9% | 20.8% | **0.026** |
| 46 to 55 | 26.2% | 24.0% | 0.105 |
| Over 56 | 6.7% | 6.5% | 0.753 |
| Do not wish to answer/missing data | 17.1% | 20.0% | 0.141 |
| **Number of years working in a nursing home** | | | |
| Less than 11 months | 10.4% | 9.2% | 0.432 |
| 1 to 5 years | 23.8% | 27.0% | 0.089 |
| 6 to 10 years | 18.3% | 16.0% | 0.928 |
| 11 years or more | 24.5% | 22.8% | 0.339 |
| Do not wish to answer/missing data | 23.0% | 25.0% | 0.390 |
| **Weekly working hours in a nursing home** | | | |
| 15 hours or fewer | 6.4% | 4.4% | **0.007** |
| 16 to 24 hours | 6.1% | 5.0% | 0.124 |
| 25 to 35 hours | 47.0% | 46.1% | 0.652 |
| 36 hours or more | 15.2% | 16.4% | 0.315 |
| Do not wish to answer/missing data | 25.2% | 28.2% | 0.141 |
