# Supplementary material for: Safety culture in French nursing homes: A randomised controlled study to evaluate the effectiveness of a risk management intervention associated with care
Source: PLoS One. 2022 Dec 1;17(12):e0277121. doi: 10.1371/journal.pone.0277121 (PMC9714758; doi:10.1371/journal.pone.0277121)
Supplement: S2 Table — (DOC) [file pone.0277121.s002.doc]

# Supplementary Material 2

Table S2: Indicators to assess the global fit of the final structural equation model on 2017 data (R output).

| **npar** | **fmin** | ***Χ*^2^** | **df** | **p-value** | **baseline. *Χ*^2^** |
| --- | --- | --- | --- | --- | --- |
| 65.000 | 0.227 | 452.186 | 188.000 | 0.000 | 10376.091 |
| baseline.df | baseline.pvalue | cfi | tli | nnfi | rfi |
| 231.000 | 0.000 | 0.974 | 0.968 | 0.968 | 0.946 |
| nfi | pnfi | ifi | rni | logl | unrestricted.logl |
| 0.956 | 0.778 | 0.974 | 0.974 | −22577.071 | −22350.751 |
| aic | bic | ntotal | bic2 | rmsea | rmsea.ci.lower |
| 45284.143 | 45602.886 | 996.000 | 45396.443 | 0.038 | 0.033 |
| rmsea.ci.upper | rmsea.pvalue | rmr | rmr_nomean | srmr | srmr_bentler |
| 0.042 | 1.000 | 0.022 | 0.022 | 0.031 | 0.031 |
| srmr_bentler_nomean | crmr | crmr_nomean | srmr_mplus | srmr_mplus_nomean | cn_05 |
| 0.031 | 0.033 | 0.033 | 0.031 | 0.031 | 487.273 |
| cn_01 | gfi | agfi | pgfi | mfi | ecvi |
| 520.358 | 0.960 | 0.946 | 0.713 | 0.876 | 0.585 |

Cronbach’s α-coefficients for the seven dimensions retained.

| **Dimensions (corresponding factors)** | **Cronbach’s α** |
| --- | --- |
| Overall perceptions of resident safety - organizational learning | 0.871 |
| Handoffs | 0.815 |
| Teamwork | 0.838 |
| Supervisor expectations and actions promoting resident safety | 0.771 |
| Compliance with procedures | 0.712 |
| Staffing | 0.729 |
| Feedback and communication about incidents | 0.731 |
