# Supplementary material for: Safety culture in French nursing homes: A randomised controlled study to evaluate the effectiveness of a risk management intervention associated with care
Source: PLoS One. 2022 Dec 1;17(12):e0277121. doi: 10.1371/journal.pone.0277121 (PMC9714758; doi:10.1371/journal.pone.0277121)
Supplement: S3 Table — (DOCX) [file pone.0277121.s003.docx]

# Supplementary Material 3

**Table S3 : Details of univariate and multivariate models explaining the evolution of safety culture scores for the 7 dimensions according to NH parameters and the implementation of the system (n=28 NH)**

|  |  | **Dimension 1** | | **Dimension 2** | | **Dimension 3** | | **Dimension 4** | | **Dimension 6** | **Dimension 7** |
| --- | --- | --- | --- | --- | --- | --- | --- | --- | --- | --- | --- |
|  |  | *Univariate* | *Multivariate* | *Univariate* | *Multivariate* | *Univariate* | *Multivariate* | *Univariate* | *Multivariate* |  |  |
| Beds | < 80 | REF. |  | REF. |  | REF. |  | REF. |  |  |  |
|  | ≥ 80 | 0,44 [-6,26;7,13] |  | 3,66 [-2,55;9,87] |  | -3,98 [-16,26;8,29] |  | 0,77 [-10,24;11,78] |  |  |  |
| Legal status | Private ou public, independant or regional | REF. |  | REF. |  | REF. |  | REF. |  |  |  |
|  | Attached to a public hospital | 0,42 [-7,29;8,14] |  | 0,48 [-6,85;7,81] |  | 7,94 [-5,98;21,86] |  | **14,58 [3,2;25,96]*** |  |  |  |
| Part of a group | Yes | REF. |  | REF. |  | REF. |  | REF. |  |  |  |
|  | No | **9,61 [2,44;16,78]*** |  | 2,8 [-4,82;10,41] |  | 1,19 [-13,42;15,79] |  | 3,85 [-8;15,7] | 5,35 [-4,74;15,43] |  |  |
|  | Hospital-based | 4,08 [-3,4;11,56] |  | 1,54 [-6,4;9,49] |  | 8,39 [-6,85;23,63] |  | **16,05 [3,68;28,41]*** | **15,53** **[2,91;28,15]*** |  |  |
| Dependency score (2016) |  | 0 [-0,05;0,05] |  | 0,01 [-0,04;0,05] |  | -0,05 [-0,13;0,03] |  | -0,01 [-0,08;0,06] |  |  |  |
| Staff/ resident ratio (2016) |  | -6,72 [-24;10,57] |  | 12,27 [-3,65;28,2] |  | 6,02 [-26,18;38,21] |  | 3,1 [-25,61;31,81] |  |  |  |
| Unqualified RM/Quality officer | No | REF. |  | REF. |  | REF. |  | REF. |  |  |  |
|  | Yes | 2,41 [-5,24;10,07] |  | -0,69 [-8,01;6,64] |  | 6,73 [-7,28;20,74] |  | -3,25 [-15,87;9,37] |  |  |  |
| Qualified RM/Quality specialist | No | REF. |  | REF. |  | REF. |  | REF. |  |  |  |
|  | Yes | 5,37 [-1,05;11,8] |  | 2,28 [-4,08;8,63] |  | 2,67 [-9,76;15,09] |  | 6,97 [-3,8;17,74] |  |  |  |
| External RM/Quality service provider | No | REF. |  | REF. |  | REF. |  | REF. |  |  |  |
| Established policy of ongoing improvement in Quality and RM | No | REF. |  | REF. |  | REF. |  | REF. |  |  |  |
|  | Yes | -1,92 [-9,03;5,19] |  | 4,01 [-2,61;10,63] |  | 7,43 [-5,47;20,33] |  | 4,17 [-7,48;15,82] |  |  |  |
| Active Quality improvement approach | No | REF. |  | REF. |  | REF. |  | REF. |  |  |  |
|  | Yes | 9,07 [0,18;17,95] | **9,58 [0,89;18,27]*** | 6,33 [-2,41;15,07] |  | 6,39 [-11,07;23,85] |  | 6,88 [-8,59;22,35] |  |  |  |
| Active RM approach | No | REF. |  | REF. |  | REF. |  | REF. |  |  |  |
|  | Yes | -3,96 [-10,63;2,71] |  | 0,75 [-5,75;7,24] |  | 7,43 [-4,87;19,73] |  | 2,25 [-8,96;13,46] |  |  |  |
| Change in the Staff/ resident ratio |  | -30,14 [-79,01;18,74] |  | 11,45 [-31,12;54,01] |  | 45,36 [-42,63;133,35] |  | 3,82 [-68,16;75,79] |  |  |  |
| Change in the dependency score |  | 0,04 [-0,07;0,15] |  | 0 [-0,11;0,1] |  | 0,09 [-0,11;0,3] |  | 0,02 [-0,14;0,17] |  |  |  |
| Unexpected disruptive events | Yes | REF. |  | REF. |  | REF. |  | REF. |  |  |  |
|  | No | 4,69 [-2,85;12,23] |  | 1,71 [-5,54;8,96] |  | 2,24 [-11,68;16,16] |  | 1,72 [-8,86;12,29] |  |  |  |
| % staff present at awareness-raising session |  | 0,05 [-0,1;0,2] |  | -0,01 [-0,16;0,13] |  | 0,01 [-0,26;0,29] |  | 0,03 [-0,21;0,28] |  |  |  |
| % staff showing leadership in the RM approach |  | 0,13 [-0,01;0,27] | **0,14 [0,01;0,28]*** | 0,04 [-0,1;0,18] |  | 0,07 [-0,2;0,35] |  | 0,12 [-0,08;0,33] |  |  |  |
| % staff wanting to use the knowledge they have gained |  | -0,01 [-0,3;0,28] |  | -0,06 [-0,32;0,2] |  | 0,34 [-0,17;0,85] |  | -0,32 [-0,7;0,06] | **-0,49 [-0,87;-0,11]*** |  |  |
| Top management attended awareness-raising session | No | REF. |  | REF. |  | REF. |  | REF. |  |  |  |
|  | Yes | 1,22 [-5,6;8,04] |  | 2,4 [-4,04;8,83] |  | -4,14 [-16,68;8,39] |  | -4,14 [-15,27;6,99] |  |  |  |
| Mature RM steering group | No | REF. |  | REF. |  | REF. |  | REF. |  |  |  |
|  | Yes | -2,44 [-9,73;4,86] |  | 2,16 [-4,5;8,83] |  | 9,84 [-3;22,68] | **9,84 [-2,2;21,87]** | 1,13 [-9,07;11,34] |  |  |  |
| Turnover among RM steering group members | No | REF. |  | REF. |  | REF. |  | REF. |  |  |  |
|  | Yes | -2,74 [-11,76;6,28] |  | -6,71 [-14,56;1,14] |  | 4,85 [-11,61;21,31] |  | 0,94 [-11,66;13,55] |  |  |  |
| % of variance explained (Adjusted R²) | |  | **20,7%** |  | **NA** |  | **16,4%** |  | **19,4%** |  |  |

**Table A3 (suite)**

|  |  | **Dimension 5** | | **Dimension 6** | | **Dimension 7** | |
| --- | --- | --- | --- | --- | --- | --- | --- |
|  |  | *Univariate* | *Multivariate* | *Univariate* | *Multivariate* | *Univariate* | *Multivariate* |
| Beds | < 80 | REF. |  | REF. |  | REF. |  |
|  | ≥ 80 | -1,81 [-12,42;8,81] |  | -3,22 [-11,28;4,83] |  | -0,73 [-6,01;4,55] |  |
| Legal status | Private ou public, independant or regional | REF. |  | REF. |  | REF. |  |
|  | Attached to a public hospital | -0,8 [-13,05;11,44] |  | 2,63 [-6,7;11,97] |  | 4,06 [-1,83;9,95] |  |
| Part of a group | Yes | REF. |  | REF. |  | REF. |  |
|  | No | 7,58 [-4,93;20,09] |  | **13,19 [4,86;21,51]*** |  | 0,13 [-6,06;6,31] |  |
|  | Hospital-based | 2,08 [-10,97;15,14] |  | 7,66 [-1,03;16,34] |  | 4,11 [-2,35;10,56] |  |
| Dependency score (2016) | | -0,03 [-0,09;0,04] |  | -0,03 [-0,09;0,02] |  | 0,01 [-0,03;0,04] |  |
| Staff/ resident ratio (2016) | | 3,43 [-24,29;31,16] |  | -12,87 [-33,55;7,82] |  | -2,61 [-16,38;11,16] |  |
| Unqualified RM/Quality officer | No | REF. |  | REF. |  | REF. |  |
|  | Yes | 1,19 [-11,06;13,43] |  | 1,25 [-8,12;10,63] |  | 0,57 [-5,52;6,66] |  |
| Qualified RM/Quality specialist | No | REF. |  | REF. |  | REF. |  |
|  | Yes | 4,61 [-5,96;15,18] |  | **8,7 [1,2;16,2]*** |  | 2,65 [-2,58;7,88] |  |
| External RM/Quality service provider | No | REF. |  | REF. |  | REF. |  |
| Established policy of ongoing improvement in Quality and RM | No | REF. |  | REF. |  | REF. |  |
|  | Yes | -2,8 [-14,11;8,51] |  | -3,38 [-11,98;5,23] |  | 2,98 [-2,56;8,51] |  |
| Active Quality improvement approach | No | REF. |  | REF. |  | REF. |  |
|  | Yes | 5,03 [-10,01;20,06] |  | **15,08 [5,01;25,14]*** | **12,02 [4,46;19,59]*** | **10,1 [3,64;16,57]*** |  |
| Active RM approach | No | REF. |  | REF. |  | REF. |  |
|  | Yes | -1,77 [-12,62;9,07] |  | 0,16 [-8,16;8,48] |  | 1,19 [-4,19;6,57] |  |
| Change in the Staff/ resident ratio |  | -35,27 [-114,18;43,64] |  | -34,7 [-97,95;28,55] |  | -10,39 [-50,97;30,2] |  |
| Change in the dependency score |  | 0,06 [-0,07;0,19] |  | 0,13 [0,03;0,24]* | **0,09 [0,01;0,18]*** | -0,03 [-0,11;0,05] |  |
| Unexpected disruptive events | Yes | REF. |  | REF. |  | REF. |  |
|  | No | **12,74 [0,82;24,66]*** | **11,1 [-0,41;22,61]** | 5,01 [-4,81;14,83] |  | 4,73 [-1,1;10,55] |  |
| % staff present at awareness-raising session |  | 0,04 [-0,2;0,28] |  | 0,01 [-0,18;0,19] |  | 0,06 [-0,06;0,17] |  |
| % staff showing leadership in the RM approach |  | 0,23 [0,01;0,44] |  | **0,21 [0,04;0,37]*** |  | -0,02 [-0,14;0,1] |  |
| % staff wanting to use the knowledge they have gained |  | 0,13 [-0,31;0,58] |  | **0,39 [0,07;0,71]*** | **0,25 [0,03;0,47]*** | -0,09 [-0,31;0,14] |  |
| Top management attended awareness-raising session | No | REF. |  | REF. |  | REF. |  |
|  | Yes | 6,51 [-4,06;17,08] |  | 0,38 [-7,94;8,71] |  | -2,18 [-7,52;3,16] |  |
| Mature RM steering group | No | REF. |  | REF. |  | REF. |  |
|  | Yes | -2,3 [-13,69;9,09] | 0,22 [-0,02;0,46] | **-8,99 [-17,27;-0,72]*** |  | 0,03 [-5,71;5,76] |  |
| Turnover among RM steering group members | No | REF. |  | REF. |  | REF. |  |
|  | Yes | -5,99 [-19,89;7,92] |  | -2,21 [-13,31;8,89] |  | -4,12 [-11;2,77] |  |
| % of variance explained (Adjusted R²) | |  | **21,2%** |  | **54,1%** |  | **NA** |

NA: No answer RM: risk management
