# Supplementary material for: Safety culture in French nursing homes: A randomised controlled study to evaluate the effectiveness of a risk management intervention associated with care
Source: PLoS One. 2022 Dec 1;17(12):e0277121. doi: 10.1371/journal.pone.0277121 (PMC9714758; doi:10.1371/journal.pone.0277121)
Supplement: Attachment — Submitted filename: PONE-D-22-13984.pdf [file pone.0277121.s004.pdf]

PLOS ONE

Safety culture in French nursing homes: a randomised controlled study to evaluate the effectiveness of a risk management intervention associated with care
The EHPAGE study

--Manuscript Draft--

Manuscript Number:	PONE-D-22-13984
Article Type:	Research Article
Full Title:	Safety culture in French nursing homes: a randomised controlled study to evaluate the effectiveness of a risk management intervention associated with care<br>The EHPAGE study
Short Title:	the effectiveness of a risk management intervention associated with care
Corresponding Author:	Noémie Terrien<br>CHU Nantes: Centre Hospitalier Universitaire de Nantes<br>Nantes, FRANCE
Keywords:	nursing homes; safety culture; survey; intervention; measurement; risk management
Abstract:	Summary<br><br>Background<br><br>French nursing homes (NHs) are in the early stages of implementing their Risk Management (RM) approach. A regional structure, which was mandated to provide independent support in RM, designed a training package.<br><br>Objective<br><br>To study the impact of the RM training package on safety culture (SC) in NHs and drivers for improvement in SC scores.<br><br>Method<br><br>This randomised controlled study targeted NHs from two French administrative divisions. Inclusion criteria were voluntary participation, no external support provided on the topic of adverse incidents upstream of the project, and the commitment of top management to its implementation. The 61 NHs were randomly allocated to one of two groups: the first benefited from a training package; support was given to the second after the impact measurement. Seven dimensions of SC were measured, at an 18-month interval, using the validated NFSOPS questionnaire (22 items) which was administered to all of the professionals working in NHs. Eleven variables were captured, relating to the structural profile of the NH, the strategic choices of top management in terms of healthcare safety, and the implementation of the system. Further modelling identified predictive factors for changes in SC scores between the two measurement times.<br><br>Results<br><br>95% of NHs completed both rounds of the questionnaire. After the intervention, the dimension Feedback and communication about incidents significantly improved (+2.8%; p-value =0.044). Improvement in the dimension Overall perceptions of resident safety – organizational learning was close to significant (+3.1%; p -value=0.075). Drivers for improvement in SC scores were a pre-existing Quality improvement approach, and a steering group that showed RM leadership.<br><br>Conclusions<br><br>The system was well-received, and appears to have improved several dimensions of SC in NHs. Our findings are all the more important given the current crisis in the healthcare sector.

Powered by Editorial Manager® and ProduXion Manager® from Aries Systems Corporation

Order of Authors:	Delphine Teigné<br>Guillaume Mabileau<br>Marion Lucas<br>Leila Moret<br>Noémie Terrien
Additional Information:	
Question	Response
**Financial Disclosure**<br><br>Enter a financial disclosure statement that describes the sources of funding for the work included in this submission. Review the submission guidelines for detailed requirements. View published research articles from *PLOS ONE* for specific examples.<br><br>This statement is required for submission and **will appear in the published article** if the submission is accepted. Please make sure it is accurate.<br><br>**Unfunded studies**<br>Enter: *The author(s) received no specific funding for this work.*<br><br>**Funded studies**<br>Enter a statement with the following details:<br>• Initials of the authors who received each award<br>• Grant numbers awarded to each author<br>• The full name of each funder<br>• URL of each funder website<br>• Did the sponsors or funders play any role in the study design, data collection and analysis, decision to publish, or preparation of the manuscript?<br>• **NO** - Include this sentence at the end of your statement: *The funders had no role in study design, data collection and analysis, decision to publish, or preparation of the manuscript.*<br>• **YES** - Specify the role(s) played.<br><br>* typeset	This study is part of the EPHAGE French research project on care system performance. The project was coordinated by QualiREL Santé, and funded by the Direction Général de l'Offre des Soins (DGOS ; https://solidarites-sante.gouv.fr/ministere/organisation/organisation-des-directions-et-services/article/organisation-de-la-direction-generale-de-l-offre-de-soins-dgos) over the period 2015–2017. The funders had no role in study design, data collection and analysis, decision to publish, or preparation of the manuscript.
**Competing Interests**<br><br>Use the instructions below to enter a competing interest statement for this submission. On behalf of all authors,	The authors have declared that no competing interests exist.

Powered by Editorial Manager® and ProduXion Manager® from Aries Systems Corporation

disclose any competing interests that could be perceived to bias this work—acknowledging all financial support and any other relevant financial or non-financial competing interests.<br><br>This statement is required for submission and **will appear in the published article** if the submission is accepted. Please make sure it is accurate and that any funding sources listed in your Funding Information later in the submission form are also declared in your Financial Disclosure statement.<br><br>View published research articles from *PLOS ONE* for specific examples.<br><br>**NO authors have competing interests**<br><br>Enter: *The authors have declared that no competing interests exist.*<br><br>**Authors with competing interests**<br><br>Enter competing interest details beginning with this statement:<br><br>*I have read the journal's policy and the authors of this manuscript have the following competing interests: [insert competing interests here]*<br><br>* typeset	
**Ethics Statement**<br><br>Enter an ethics statement for this submission. This statement is required if the study involved:<br><br>• Human participants<br>• Human specimens or tissue<br>• Vertebrate animals or cephalopods<br>• Vertebrate embryos or tissues<br>• Field research<br><br>Write "N/A" if the submission does not require an ethics statement.	The EHPAGE research protocol was approved by the Advisory Committee on Health Research Information Processing, and an ethics committee (Groupe Nantais d'Ethique dans le Domaine de la Santé [University Hospital of Nantes]). The participation of professionals in the research was the subject of prior information. Professionals were informed about the purpose of the research in advance. Participation was voluntary and informed consent was implied upon completion of the questionnaire (a procedure approved by the ethics committee). According to articles L1121-1 and R1121-2 of the French Public Health Code, Institutional Review Board approval was not necessary.

Powered by Editorial Manager® and ProduXion Manager® from Aries Systems Corporation

General guidance is provided below. Consult the submission guidelines for detailed instructions. **Make sure that all information entered here is included in the Methods section of the manuscript.**<br><br>**Format for specific study types**<br><br>**Human Subject Research (involving human participants and/or tissue)**<br>• Give the name of the institutional review board or ethics committee that approved the study<br>• Include the approval number and/or a statement indicating approval of this research<br>• Indicate the form of consent obtained (written/oral) or the reason that consent was not obtained (e.g. the data were analyzed anonymously)<br><br>**Animal Research (involving vertebrate animals, embryos or tissues)**<br>• Provide the name of the Institutional Animal Care and Use Committee (IACUC) or other relevant ethics board that reviewed the study protocol, and indicate whether they approved this research or granted a formal waiver of ethical approval<br>• Include an approval number if one was obtained<br>• If the study involved *non-human primates*, add *additional details* about animal welfare and steps taken to ameliorate suffering<br>• If anesthesia, euthanasia, or any kind of animal sacrifice is part of the study, include briefly which substances and/or methods were applied<br><br>**Field Research**<br><br>Include the following details if this study involves the collection of plant, animal, or other materials from a natural setting:<br>• Field permit number<br>• Name of the institution or relevant body that granted permission	
**Data Availability**<br><br>Authors are required to make all data	Yes - all data are fully available without restriction

Powered by Editorial Manager® and ProduXion Manager® from Aries Systems Corporation

underlying the findings described fully available, without restriction, and from the time of publication. PLOS allows rare exceptions to address legal and ethical concerns. See the PLOS Data Policy and FAQ for detailed information.<br><br>A Data Availability Statement describing where the data can be found is required at submission. Your answers to this question constitute the Data Availability Statement and **will be published in the article**, if accepted.<br><br>**Important:** Stating 'data available on request from the author' is not sufficient. If your data are only available upon request, select 'No' for the first question and explain your exceptional situation in the text box.<br><br>Do the authors confirm that all data underlying the findings described in their manuscript are fully available without restriction?	
**Describe where the data may be found in full sentences. If you are copying our sample text, replace any instances of XXX with the appropriate details.**<br><br>• If the data are **held or will be held in a public repository**, include URLs, accession numbers or DOIs. If this information will only be available after acceptance, indicate this by ticking the box below. For example: *All XXX files are available from the XXX database (accession number(s) XXX, XXX.).*<br>• If the data are all contained **within the manuscript and/or Supporting Information files**, enter the following: *All relevant data are within the manuscript and its Supporting Information files.*<br>• If neither of these applies but you are able to provide **details of access elsewhere**, with or without limitations, please do so. For example:<br><br>*Data cannot be shared publicly because of [XXX]. Data are available from the*	All relevant data are within the manuscript and its Supporting Information files.

XXX Institutional Data Access / Ethics Committee (contact via XXX) for researchers who meet the criteria for access to confidential data.<br><br>*The data underlying the results presented in the study are available from (include the name of the third party and contact information or URL).*<br>• This text is appropriate if the data are owned by a third party and authors do not have permission to share the data.<br><br>* typeset	
Additional data availability information:	Tick here if the URLs/accession numbers/DOIs will be available only after acceptance of the manuscript for publication so that we can ensure their inclusion before publication.

Powered by Editorial Manager® and ProduXion Manager® from Aries Systems Corporation

Manuscript
Click here to access/download;Manuscript;ArticlePrincepsCStraductionMAI

Safety culture in French nursing homes: a randomised controlled study to evaluate the effectiveness of a risk management intervention associated with care

The EHPAGE study

Delphine Teigné[a,b], Guillaume Mabileau[a], Marion Lucas[a], Leila Moret[a,b,c], Noémie Terrien[a*]

Delphine Teigné: 0000-0001-8179-4188

Leila Moret: 0000-0003-1285-6394

Noémie Terrien: 0000-0001-8383-6804

[a] QualiREL Santé, Hôpital Saint Jacques, 85 rue Saint Jacques, 44093 NANTES

[b] Public Health Department, University Hospital of Nantes, 85 rue Saint-Jacques, 44093 Nantes Cedex 1, France

[c] UMR INSERM U1246-SPHERE "methodS for Patients-centered outcomes & HEalth REsearch", University of Nantes, University of Tours, 22 Boulevard Benoni Goullin 44000 Nantes, France

Email addresses

DT: operative project leader; delphine.teigne@chu-nantes.fr

GM: statistician; gmabileau@qualirelsante.com

ML: project manager assistant; mlucas@qualirelsante.com

LM: M.D. PhD; leila.moret@chu-nantes.fr

NT*: delegated investigator; corresponding author: nterrien@qualirelsante.com; tel: 02 40 84 69 31; QualiREL Santé, Hôpital Saint Jacques, 85 rue Saint Jacques, 44093 NANTES

Keywords: nursing homes, safety culture, survey, intervention, measurement, risk management

Summary

Background: French nursing homes (NHs) are in the early stages of implementing their Risk Management (RM) approach. A regional structure, which was mandated to provide independent support in RM, designed a training package.

Objective: To study the impact of the RM training package on safety culture (SC) in NHs and drivers for improvement in SC scores.

Method: This randomised controlled study targeted NHs from two French administrative divisions. Inclusion criteria were voluntary participation, no external support provided on the topic of adverse incidents upstream of the project, and the commitment of top management to its implementation. The 61 NHs were randomly allocated to one of two groups: the first benefited from a training package; support was given to the second after the impact measurement. Seven dimensions of SC were measured, at an 18-month interval, using the validated NFSOPS questionnaire (22 items) which was administered to all of the professionals working in NHs. Eleven variables were captured, relating to the structural profile of the NH, the strategic choices of top management in terms of healthcare safety, and the implementation of the system. Further modelling identified predictive factors for changes in SC scores between the two measurement times.

Results: 95% of NHs completed both rounds of the questionnaire. After the intervention, the dimension *Feedback and communication about incidents* significantly improved (+2.8%; *p-value*=0.044). Improvement in the dimension *Overall perceptions of resident safety* –

organizational learning was close to significant (+3.1%; *p*-value=0.075). Drivers for improvement in SC scores were a pre-existing Quality improvement approach, and a steering group that showed RM leadership.

Conclusions: The system was well-received, and appears to have improved several dimensions of SC in NHs. Our findings are all the more important given the current crisis in the healthcare sector.

Trial registration: Retrospectively registered as NCT02908373 (September 21, 2016).

Introduction

Faced with an ageing world population and an increase in chronic disease, health systems are constantly evolving [1]. In France, the accommodation provided to dependent elderly people who require day-to-day care has undergone many changes. French nursing homes (NHs) accommodate elderly people who have lost their physical and/ or mental independence, and who can no longer live in their own home [2]. Residents are scored according to their degree of dependency [3]. The establishment offers assistance in daily life (getting up and going to bed, washing, meals, etc.) and services such as catering, laundry and entertainment. Medical and paramedical care is either provided by staff who are employed directly by the NH (care assistants, nurses, etc.) or by external professionals who are contracted (local doctors, physiotherapists, chiropodists, etc.). The legal status of French NHs can vary (independent, part of a group, hospital-based). They also differ in terms of the number of beds, number of staff, and the ratio of staff to residents. But all of them have to address problems associated with the specific nature of the population they serve, and overcome organizational and financial difficulties [3].

At the national level, the French Patient Safety Programme 2013–2017 focused on the fight against the occurrence of adverse events associated with care (AEAC) using a Risk Management (RM) approach [4]. The programme's report highlighted that there was a long way to go before French NHs implement the RM approach [5], and emphasized the importance of adapting the AEAC concept to the specific context of the day-to-day care and assistance provided in the place of residence [2].

In general, since the 2010s, approaches to improving the quality and safety of care have included the development of a Safety Culture (SC) among professionals [6]. SC is a multidimensional concept, and there is currently no consensus on the definition, number, nature and denomination of its dimensions [7]. The European Society for Quality in Health Care defines it as, “an integrated pattern of individual and organizational behavior, based upon shared beliefs and values that continuously seeks to minimize patient harm, which may result from the processes of care delivery” [8]. James Reason argues that a SC can play a key role in preventing incidents and, therefore, improve the safety of care [7]. The assessment of SC helps to raise awareness of patient safety issues, and identify potential areas for improvement.

According to Shortell et al., it is one of the key pillars for achieving quality and RM objectives [9]. In this regard, various tools have been developed to measure it. In particular, the American Agency for Healthcare Research and Quality has developed a questionnaire tailored to NHs: the Nursing Home Survey on Patient Safety Culture (NHSOPS) [10]. Its adaptation to different contexts has been the subject of various articles [11–13], including a study in France [14]. The French version of the psychometrically-validated (NFSOPS-F) includes 22 items that measure seven dimensions of SC.

In 2016, the French government created several Regional Support Structures (RSS) spread throughout the country. The main role of these structures is to support healthcare professionals in hospitals and medico-social establishments (including NHs) by providing expertise on quality improvement and patient care safety [15]. Only a few examples of

support systems for healthcare professionals have been reported in the literature, and they mainly concern team training, the creation of patient safety teams, or patient safety education programs implemented in hospitals—and even fewer have been evaluated for their effectiveness [16]. Moreover, few target NHs [17]or care at home [18].

As, to the best of our knowledge, there are no reports of schemes that propose a comprehensive RM structure tailored to NHs, QualiREL Santé (a RSS for Quality and Safety of Care) designed a training package to support French NHs in the implementation of their RM system. The scheme aimed to provide a long-term strategy for structuring and reporting AEAC of different levels of severity, and was based on the four axes found in the Shortell model [9]. It targeted staff at all hierarchical levels, whatever their role in supporting the elderly person. The aim was to develop SC among professionals and, more specifically, improve overall perceptions of resident safety. It was therefore important to study the impact of the implementation of the intervention on SC.

Any recommendations for improvements to the support system, or its successful implementation, must be underpinned by an understanding of the parameters that influence the final results [19–21]. These parameters may be related to the structural profile of the NH, its appropriation of the system, or the strategic healthcare safety choices made by top management [22].

The main objective of our research was, therefore, to study the impact of the implementation of an RM support system, which was deployed as training package, on the SC of professionals. We measured the evolution of SC in a group of NHs supported in their

RM strategy; and the relation between the change in SC scores and the characteristics of the NH. Our results provide an insight into the actions and strategies that could be adopted to develop SC in NHs.

The present study is part of the EHPAGE research project, which looked at the performance of the French healthcare system, and was financed by the General Directorate for Healthcare Provision (*Direction générale de l'offre de soins*) (2015–2017).

Method

Participating nursing homes

A total of 61 NHs from two French administrative divisions (*départements*) (Loire-Atlantique and Vendée) participated. Inclusion criteria were: i) voluntary participation in September 2015 to benefit from a system to support the implementation of a RM approach, ii) a written commitment by the NH's top management to implement a RM approach; and iii) no external support had been provided by QualiREL Santé on the topic of adverse incidents upstream of the project.

Tools

The training package

The overall objective of the training package was to outline a strategy to structure the reporting and analysis of AEAC in the long term. In particular, the goals were to: i) provide a definition of AEAC tailored to the context of French NHs, with illustrative examples [2]; ii) to

propose scales for the severity and frequency of AEAC [23] ; iii) to provide formal tools for a reporting and analysis system; and, iv) to create a steering group of NH professionals that would be responsible for the analysis of AEACs and the definition of an action plan.

The training package was expected to lead to improvements in SC among NH staff, particularly overall perceptions of resident safety. It consisted of 20 hours (for each NH) of methodological support provided by two facilitators from the research team. Support was structured into four interventions. The initial session (1) raised awareness among top management of the importance of taking ownership of the project. The aim of the second session (2) was to raise awareness among all NH staff of the need to report AEAC, and identify volunteers who would join the steering group. The third session (3) was run for members of the RM steering group; here the aim was to train them in how to structure their work and analyze AEAC. Then, the aim of the final session (4) was to observe how the steering group handled a critical AEAC that had occurred in the NH, and provide support in its analysis. At the end of each intervention, dedicated tools that had been specifically designed for the package were presented, and made available to participants. Table 1 presents the training package as a function of the four axes in Shortell's model [9]. The four sessions were implemented over a period of nine months.

PLEASE INSERT TABLE 1

Table 1. Presentation of the training package categorized according to the 4 axes in Shortell's model.

**Axes in Shortell's model** [9]	**Support provided by QualiREL Santé**	**Targeted themes**	**Tools provided to the NH**
**Strategic**	Session 1	• Raise the awareness of top managers of the importance of the project's implementation (challenges are included in the establishment's policy, definition of objectives in the Nursing Home's Quality-Risk Program).	• Project implementation commitment charter signed by top management.
**Cultural**	Sessions 2 and 3	• Raising awareness of the value of reporting.<br>• Raising awareness of a positive culture of error.<br>• Raising awareness of the importance of feedback.	• Sample charter to encourage AEAC reporting.<br>• Communication support, including feedback.
**Structural**	Session 1	• Identification of a peer as a contact point for the project	
	Session 3	• Setting up a RM steering group.	• Template operating charter for the RM steering group.<br>• Template procedure for reporting AEACs.<br>• Template AEAC report form.
**Technical**	Session 3	• Train members of the RM steering group in the monitoring and analysis of AEACs.	• Computerized database for recording and monitoring AEACs.
	Session 4	• Provide support during the first analysis.	

AEAC: adverse events associated with care RM: Risk Management

Our recommendations for the implementation of the training package were as follows: as many NH staff (including top management) as possible should attend the awareness-raising session; the RM steering group should be composed of different staff categories (nurses, local doctors, care assistants, staff responsible for catering and laundry, secretaries, etc.); and the steering group should have met at least once (and, if possible, should have run an analysis of an AEAC that had occurred in the NH) before taking part in the fourth session.

The Nursing Home Survey on Patient Safety Culture

The French version of the psychometrically-validated NHSOPS includes 22 items that measure the following seven dimensions of SC [14]. Dimension 1 (*Overall perceptions of resident safety and organizational learning*) explores the ability of the facility to take account of its mistakes and make changes, and the overall quality of care provided for residents. Dimension 2 (*Handoffs*) assesses communication for resident care both within the NH, and with external structures. Dimension 3 (*Teamwork*) explores support, mutual respect and collaboration among colleagues. Dimension 4 (*Supervisor expectations and actions promoting resident safety*) captures supervisors' acknowledgement of compliance with procedures, and their openness to ideas for improvement. Dimension 5 (*Compliance with procedures*) explores general compliance with mandatory procedures implemented in the facility, regardless of any operational difficulties encountered by professionals. Dimension 6 (*Staffing*) captures the number of staff in relation to the workload. Finally, Dimension 7 (*Feedback and communication about incidents*) explores how potential or actual incidents are dealt with, and solutions are shared.

This self-administered questionnaire is aimed at professionals working in French NHs (both staff employed directly by the NH, and external contracted professionals). It is divided into four sections: “Working in your NH”, “Communication”, “Your hierarchy” and “Your NH”. There are 22 items, which are scored on a 5-point Likert scale (*never*, *rarely*, *sometimes*, *most of the time, always* – or – *do not agree at all*, *do not agree*, *agree up to a point*, *agree*, *completely agree*). “Does not apply” and “Don’t know” are other response categories. Finally, the questionnaire includes items relating to the sociodemographic characteristics of respondents.

Study design

The study followed the randomised controlled study design. It ran from September 2015 to November 2017.

Randomization

The 61 NHs were randomly allocated into two groups; the first group received support in 2016 (experimental group – 31 NHs), and the second group received support after the impact assessment in November 2017 (control group – 30 NHs). The homogeneity of the two groups in terms of distribution by *département* and legal status was checked.

Implementation of the training package

The operational implementation of the support system ran over a period of nine months. Two facilitators from the research team spent time in each NH. The latter had a training and

coaching role in the implementation of the support system (from sessions 1 to 4). Two professionals were appointed by the NH to be the main point of contact during the project.

SC measurement

The same NFSOPS-Fs questionnaire was administered to the two groups prior to the implementation of the training package (January 2016), and again around 18 months later (September 2017). It was important that the NH took ownership of the support system over time. The methodology for the distribution and collection of questionnaires has been extensively described in an earlier article [22]. To summarize, in each NH the two contact points were responsible for: distributing the questionnaire to each professional working in the NH; promoting and following-up the survey; centralizing the completed questionnaires; and returning them to the research team [22]. The survey was conducted over a six-week period. Each completed questionnaire was given a number to ensure anonymity. As SC was measured at the level of the NH, we did not attempt to compare answers for each respondent between the two measures. Responses were recorded a by an external technician, and data capture was blind, in duplicate, with cross-checking.

Descriptive variables for NHs in the experimental group

Eleven descriptive variables were collected at the beginning of the project in 2016. Five related to the structure of the NH: *bed capacity*; *legal status*; *being part of a group or not*; the *mean Dependency score*; and *the staff/ resident ratio*. Six other variables documented top managers' strategic choices in terms of safety of care: *an established policy of ongoing*

improvement in Quality and RM; *an active Quality improvement approach*; and *an active RM approach,* approaches facilitated by the presence of either: *an appointed RM Quality officer* (a member of staff with no specialized RM Quality qualifications)*, a RM Quality specialist* (a graduate with RM Quality qualifications), or *an external Quality and RM service provider*. The categorization of these variables is presented in Table 2.

PLEASE INSERT TABLE 2

Table 2: Descriptive variables of nursing homes and the implementation of the scheme.

**Descriptive variables in 2016**	**Case group n=28 NHs**
	**n (%) or mean (SD)**
Beds*	
≥80	15 (53.5%)
<80	13 (46.5%)
Legal status *	
Private or public, independent or regional	21 (75%)
Attached to a public hospital	7 (25%)
Part of a group *	
No	13 (46.5%)
Yes	8 (53.5%)
Hospital-based	7 (25%)
Dependency score*	
Mean (SD)	625 (75.54%)
NA	1 (3.6%)
Staff/ resident ratio *	
Mean (SD)	0.74 (0.19%)
NA	0 (0.0%)
Unqualified RM/Quality officer **	
No	7 (25%)
Yes	21 (75%)
NA	0 (0%)
Qualified RM/Quality specialist **	
No	16 (57.1%)
Yes	12 (42.9%)
NA	0 (0%)
External RM/Quality service provider **	
No	28 (100%)
Yes	0 (0%)

NA	0 (0%)
Established policy of ongoing improvement in Quality and RM **	9 (32.1%)
No	19 (67.9%)
Yes	0 (0%)
NA	
Active Quality improvement approach **	
No	4 (14.3%)
Yes	24 (85.7%)
NA	0 (0%)
Active RM policy **	
No	11 (39.3%)
Yes	17 (60.7%)
NA	0 (0%)
**Change in descriptive variables for nursing homes in the experimental group in 2017**	**Case group n=28<br>n (%) or mean (SD)**
Change in the staff/ resident ratio*	
Mean (SD)	+0.03 (0.08)
NA	4 (14.3%)
Change in the dependency score*	
Mean (SD)	+14.3 (35.7)
NA	5 (17.9%)
**Variables related to the implementation of the system in the experimental group in 2017**	**Case group n=28<br>n (%) or mean (SD)**
% staff present at the awareness-raising session (session 2)**	49.5 (22.5)
Mean (SD)	0 (0.0%)
NA	
% top management present at the awareness-raising session (session 2)**	
No	11(39.3%)
Yes	17 (60.7%)
% staff wanting to use the knowledge they have gained**	
Mean (SD)	91.1 (12.6)
NA	2 (7.1%)
% staff showing leadership in the RM approach**	
Mean (SD)	34.0 (24.1)
NA	2 (7.1%)
Mature RM steering group**	
No	10 (35.7%)
Yes	16 (57.2%)
NA	2 (7.1%)
Turnover among RM steering group members**	
No	21 (75%)

Yes	5 (17.9%)
NA	2 (7.1%)
Unexpected disruptive events*	
No	15 (53.6%)
Yes	8 (28.5%)
NA	5 (17.9%)

RM: Risk Management NA: no answer SD: Standard Deviation
* Variables related to the structural profile of NHs
** Variables related to strategic safety of care choices

Change in descriptive variables for NHs in the experimental group in 2017

In 2017, change in scores for two variables related to the structural profile of the NH were captured: *the staff/ resident ratio*, and the *mean Dependency score* (Table 2).

Change in variables related to the implementation of the training package in the experimental group in 2017

As the project progressed, the following seven variables related to recommendations for implementing the system were measured.

1. The percentage of staff who attended the awareness-raising session.
2. The presence of top management at the awareness-raising session.
3. An anonymous questionnaire was distributed to each member of the steering group at the end of session 3. This questionnaire identified the percentage of professionals who “wanted to use the knowledge they had gained” or
4. who “felt able to pass on their knowledge to colleagues”.

5. The degree of maturity of the steering group in implementing the approach was recorded by the two facilitators. This measure corresponded to the use of the systemic AEAC analysis tool that was made available between the 3rd and 4th sessions.
6. Facilitators also recorded the stability of the steering group, notably the withdrawal or replacement of its members.
7. Finally, the research team noted any contextual elements that could undermine the implementation of the system (for example, the relocation of the NH, turnover among top management, absenteeism among local management, and a lack of commitment from top management).

The categorization of these seven variables is shown in Table 2. The research team developed bespoke tools to collect this data, and these tools are not licensed.

Data analysis

The calculation of SC scores has been described in detail in previous work [22]. All analyses were performed at the NH level. Following the method given in the original American version of the NHSOPS [10], scores were calculated as the average percentage of positive responses on all items in each dimension.

The validity of the model constructed from data collected from the first round of questionnaires [14] was rechecked against data from the second round. Four statistical indices were taken into account in order to verify the fit of the model to the data: Steiger's

Root Mean Square Error of Approximation (RMSEA), and the Standardized Root Mean Squared Residual (SRMR): fit was considered good when these values were <0.1, and very good when <0.05. The Tucker Lewis Index (TLI) and the Comparative Fit Index (CFI) were considered an acceptable fit when >0.9 [24]. Cronbach's α coefficients were calculated to assess internal consistency [25]. Consistency was considered acceptable when α>0.70 [26]. SC scores were calculated for each NH that completed the two questionnaires in 2016 (baseline) and in 2017 (following the intervention). Scores for the seven dimensions for each NH were compiled at the group level (experimental group = 30 NHs; control group = 28 NHs). Thresholds to discriminate the levels of development of the dimensions were set at: SC <50% (underdeveloped); 50% ≤ SC <75% (developing); and SC ≥ 75% (developed) [14].

Changes in scores for the seven dimensions were evaluated using a paired Student's *t*-test. A threshold of 0.05 was used to establish a significant difference between the two measures.

Descriptive statistics were run for all variables.

Linear regression models were used to assess variables associated with each change in the SC score in univariate analyses to identify potential predictors at a significance level of 0.2. Multivariate analyses were then performed according to the backward stepwise method, in order to identify models that best fitted the data, and keep the most relevant factors associated with each change in the SC score, at a significance level of 0.05. Validity and goodness of fit were checked using adjusted R^2 and an F-test.

All statistical analyses were performed using R software (version 4.1.1).

Ethics approval and consent to participate

The EHPAGE research protocol was approved by the Advisory Committee on Health Research Information Processing, and an ethics committee (*Groupe Nantais d'Ethique dans le Domaine de la Santé* [University Hospital of Nantes]). Professionals were informed about the purpose of the research in advance. Participation was voluntary and informed consent was implied upon completion of the questionnaire (a procedure approved by the ethics committee). According to articles L1121-1 and R1121-2 of the French Public Health Code, Institutional Review Board approval was not necessary.

Results

NH participation

Participation in the implementation of the training package

By November 2017, 30 of the 31 NHs in the intervention group had benefited from the entire support package (Fig. 1).

PLEASE INSERT FIG. 1

Fig 1 : Flow chart of the study in control and intervention groups

Participation in the NFSOPS-F questionnaire

Out of the 61 NHs operating in 2015, 58 (95%) completed the two rounds of questionnaires (Fig. 1).

The questionnaire was administered to 3,390 professionals in 2016 (1,946 responded) and 3,140 professionals in 2017 (1,621 responded). Of those who responded in 2016, 1,012 were in the experimental group and 934 were in the control group. In 2017, these figures were 754 (experimental group) and 867 (control group). The median overall participation rate for all NHs was 64% in 2016 and 56.5% in 2017. Table A1 (Supplementary Material) compares the characteristics of respondents in 2016 and 2017. The main differences between 2016 and 2017 concern: the percentage of paramedical professionals (+3.6%; *p*-value=0.038) and doctors (−1.1%; *p*-value=0.001); the percentage of professionals aged under 25 (+2.3%; *p*-value=0.002) and between 36 and 45 (−3.1%; *p*-value=0.026); and professionals working 15 hours or less per week (−2%; *p*-value=0.007). All other categories were not statistically different at the 5% alpha level.

Descriptive variables

Descriptive variables for NHs in the experimental group

Of the 28 NHs in the experimental group that completed the two questionnaires, seven were hospital-based, and 21 had another status. Bed capacity was equal to or over 80 for 15 NHs. The mean staff/ resident ratio was 0.74 in 2016 and 0.77 in 2017. The mean Dependency score was 625 in 2016 and 639.3 in 2017. In 2016, an active Quality

improvement approach was in place in 24 NHs, and there was an active RM approach in 17. For 12 NHs, these policies were facilitated by a specialized RM Quality graduate, while in 21 NHs they were the responsibility of a salaried NH professional with no specific RM Quality qualifications. These results are presented in Table 2.

Change in descriptive variables for NHs in the experimental group

The analysis found that in the 28 NHs in the experimental group, there was no significant change in the staff/ resident ratio, or the mean Dependency score during the research period (Table 2).

Variables related to the implementation of the training package

On average, 49.5% of NH professionals attended the awareness-raising session; top management were present in 60.7% of NHs. On average, 7.2 professionals were involved in the steering group, and on average, 96.2% of steering group members completed the questionnaire that was administered after session 3. The analysis found that 91.1% of steering group members wanted to put into practice the knowledge they had acquired during the training sessions, and 34% showed leadership in the RM process. Finally, following session 4, the steering group was considered mature in 57.2% of NHs, and stable in 75%. Table 2 documents the seven variables used to measure the impact of the intervention.

SC scores, group homogeneity

The validity of the model constructed in 2016 was confirmed by data from 2017 (Table A2 Supplementary Material).

Table 3 presents scores for the seven SC dimensions for experimental and control groups in 2016 and 2017. The analysis found no statistically significant differences between the two groups at the beginning of the project.

PLEASE INSERT TABLE 3

Table 3: Scores for each of the seven SC dimensions by group and survey round.

**Group** (number of NH)	**Survey number** (date)	**SC scores by dimension (%)**						
		**Dimension 1** ***Overall perceptions of resident safety - organizational learning***	**Dimension 2** ***Handoffs***	**Dimension 3** ***Teamwork***	**Dimension 4** ***Supervisor expectations and actions promoting resident safety***	**Dimension 5** ***Compliance with procedures***	**Dimension 6** ***Staffing***	**Dimension 7** ***Feedback and communication about incidents***
		mean Q1 Q3	mean Q1 Q3	mean Q1 Q3	mean Q1 Q3	mean Q1 Q3	mean Q1 Q3	mean Q1 Q3
Total (n=58 NHs)	1 (January 2016)	75.8% [69.5%; 87.8%]	76.9% [72.0%;84.2%]	60.4% [48.8%;72.4%]	79.2% [72.7%;87.4%]	55.0% [46.0%;62.9%]	11.8% [3.7%;17.7%]	84.8% [79.3%;91.9%]
Experimental (n= 28 NHs)	1 (January 2016)	75.5% [70.8%;90.3%]	78.7% [74.0%;85.9%]	60.9% [48.8%;70.5%]	77.6% [72.7%;88.8%]	56.1% [47.8%;64.1%]	11.6% [4.2%;18.4%]	85.4% [80.3%;94.6]
	2 (September 2017)	78.6% [75.0%;88.7%]	75.9% [72.1%;82.9%]	58.1% [43.5%;74.0%]	82.8% [76.4%;89.7%]	54.1% [43.1%;64.9%]	10.3% [3.0%;16.5%]	88.2% [83.7%;93.7%]
Control (n= 30 NHs)	1 (January 2016)	76.0% [69.1%;86.6%]	75.3% [70.7%;83.2%]	59.9% [51.0%;72.4%]	80.8% [77.9%;87.1%]	53.9% [44.4%;59.5%]	12.0% [3.6%;16.6%]	84.2% [79.6%;91.3%]
	2 (September 2017)	72.3% [67.2%;86.0%]	74.9% [70.3%;81.9%]	57.9% [47.3%;65.2%]	79.6% [76.5%;87.4%]	51.7% [44.4%;60.7%]	12.0% [3.4%;16.9%]	85.1% [80.5%;92.3%]

SC: Safety Culture NH: Nursing Home SC < 50%: underdeveloped 50% ≤ SC < 75%: developing SC ≥ 75%: developed

Table 4 reports changes in SC scores between the administration of the two questionnaires in experimental and control groups. There were no significant changes in the control group between the two runs. However, in the experimental group, scores for Dimension 7 (*Feedback and communication about incidents*; *p*-value=0.044) did increase significantly, indicating an improvement. Scores for two other dimensions (Dimensions 1 and 4; *Overall perceptions of resident safety - organizational learning* and *Supervisor expectations and actions promoting resident safety*) also increased, and were close to significance (*p*-values of 0.075 and 0.067) (Table 4). Finally, scores for Dimension 2 (*Handoffs*) fell, and the fall was close to significant (*p*-value of 0.089).

PLEASE INSERT TABLE 4

Table 4: Change in SC scores between 2016 and 2017 for each dimension and for each group.

**Group (number of NH)**	**% change by dimension (*p*-value)**						
	**Dimension 1** ***Overall perceptions of resident safety - organizational learning***	**Dimension 2** ***Handoffs***	**Dimension 3** ***Teamwork***	**Dimension 4** ***Supervisor expectations and actions promoting resident safety***	**Dimension 5** ***Compliance with procedures***	**Dimension 6** ***Staffing***	**Dimension 7** ***Feedback and communication about incidents***
Experimental (n=28 NHs)	3.1% (0.075)	−2.8% (0.089)	−2.8% (0.368)	5.3% (0.067)	−2.0% (0.464)	−1.3% (0.531)	2.8% (**0.044**)
Control (n=30 NHs)	−3.7% (0.266)	−0.4% (0.806)	−2.0% (0.488)	−1.2% (0.619)	−2.2% (0.427)	−0.1% (0.960)	0.9% (0.530)

SC: Safety Culture NH: Nursing Home SC < 50%: underdeveloped 50% ≤ SC < 75%: developing SC ≥ 75%: developed.

Relationships between variables and change in SC scores

The results of the final multivariate models are presented in Table 5, and details of the univariate models used to construct them are presented in Table A3 (Supplementary Material). The analysis found that scores for Dimension 1 (*Overall perceptions of resident safety - organizational learning*) improved significantly as a function of the percentage of members of the steering group who showed leadership, and in NHs with an active Quality improvement approach (respectively, β-coefficient=0.14/9.58; 95%CI=[0.01;0.28]/[0.89;18.27]). Scores for Dimension 3 (*Teamwork*) improved in cases where the NH had an established policy of ongoing improvement in Quality and RM (β-coefficient=−12.42; 95%CI=[0.71; 24.12]). Scores for Dimension 4 (*Supervisor expectations and actions promoting resident safety*) improved when the NH was hospital-based – but not when it was independent, or when fewer members of the steering group stated that they wanted to use the knowledge they had gained from the training sessions (β-coefficient=15.53/−0.49; 95%CI=[2.91;28.15]/[−0.87;−0.11]). Scores for Dimension 5 (*Compliance with procedures*) improved when no unexpected disruptive events occurred (β-coefficient=11.1; 95%CI=[−0.41;22.61]). Scores for Dimension 6 (*Staffing*) improved as a function of the percentage of members of the steering group who wanted to use the knowledge they had acquired, when there was an active Quality improvement approach, and when the Dependency score increased (β-coefficient=0.25/12.02/0.09; 95%CI = [0.03;0.47]/[4.46;19.59]/ [0.01;0.18]). The model explained 54.1% of variability in the data for Dimension 6.

PLEASE INSERT TABLE 5

Table 5: Parameters included in the multivariate models that explain change in scores for the seven dimensions of safety culture (n=28 NHs).

**Parameter**		**Coef. [IC 95%]**
**Dimension 1:** ***Overall perceptions of resident safety - organizational learning***		***Adjusted R^2 = 20.7%.***
Active Quality improvement approach	No	ref.
	Yes	9.58 [0.89;18.27]*
% staff showing leadership in the RM approach		0.14 [0.01;0.28]*
**Dimension 2:** ***Handoffs***		
N/A		
**Dimension 3:** ***Teamwork***		***Adjusted R^2 = 16.4%.***
Established policy of ongoing improvement in Quality and RM	No	ref.
	Yes	12.42 [0.71;24.12]*
Mature RM steering group	No	ref.
	Yes	9.84 [−2.2;21.87]
**Dimension 4:** ***Supervisor expectations and actions promoting resident safety***		***Adjusted R^2 = 19.4%.***
Membership of a group	No	ref.
	Yes	5.35 [−4.74;15.43]
	Hospital-based	15.53 [2.91;28.15]*
% staff wanting to use the knowledge acquired		−0.49 [−0.87;−0.11]*
**Dimension 5:** ***Compliance with procedures***		***Adjusted R^2 = 21.2%.***
Unexpected disruptive events	Yes	ref.
	RAS	11.1 [−0.41;22.61]
% staff showing leadership	No	0.22 [−0.02;0.46]
**Dimension 6:** ***Staffing***		***Adjusted R^2 = 54.1%.***
Active Quality improvement approach	No	ref.
	Yes	12.02 [4.46;19.59]*
Change in dependency score		0.09 [0.01;0.18]*
% staff wanting to use the knowledge acquired		0.25 [0.03;0.47]*
**Dimension 7:** ***Feedback and communication about incidents***		
N/A		

*Significant at 5%   **Significant at <0.1%

N/A: not analyzable   RM: Risk Management

Discussion

Change in SC scores

No change was observed in the SC of the control group, in contrast to the experimental group. Our results highlight the impact of the support system on the SC of professionals in the experimental group.

The intervention was initially designed to improve scores for Dimension 1 (*Overall perceptions of resident safety*). However, the validation of the French version of the questionnaire that was carried out during the research project led to the aggregation of two dimensions that are distinct in the American version [10]. The new Dimension 1 was therefore termed *Overall perceptions of resident safety - organizational learning*. Scores for this dimension, which were already high (75.8%) at the beginning of the project, improved (by +3.1%) after the implementation of the support system. However, the aggregation of the two dimensions made it impossible to demonstrate with certainty the impact of the system on the specific aspect of this dimension. The addition of *organizational learning* reflects a process of profound change at different levels of the NH [16]. This process has begun in the NHs that participated in the research project, and ongoing improvement in organizational learning will be continue to be measured in the next several years [7].

Improvements in two other dimensions deserve to be highlighted. First, Dimension 7 (*Feedback and communication about incidents*), which was already developed at the beginning of the project (85.4%), significantly improved (by +2.8%). This change was brought

about by, among other actions, the awareness-raising sessions, the tools that were made available, and empowering a group of professionals to analyze AEACs and provide feedback to colleagues on the action plan. Improvements in Dimension 4 (*Supervisor expectations and actions promoting resident safety*), which was also initially developed (77.6%) were close to significant (+5.3%). The formal implementation of the approach required NHs in the experimental group to change their day-to-day practices, which was only possible with the investment of top management in the project. The approach encourages top managers to listen to the suggestions and ideas put forward by staff to improve the safety of residents, and this may, in turn, have helped top managers to clearly express their expectations and acknowledge good performance.

The literature agrees that the dimensions are interlinked; thus, taking action in one dimension could have an indirect impact on the others [27]. For example, Dimension 2 (*Handoffs*), which was developed (78.7%) at baseline, decreased (by −2.8%) between the two measures, which was close to being significant. At baseline, NH staff generally agreed that the information that was made available to them (whether a new care plan or a change to an existing plan) was complete, and allowed for satisfactory patient management. However, it appears that the support provided by the research team alerted professionals to the importance of dysfunctions in this area, and possible ways to make improvements. An in-depth review by the whole NH team, and the implementation of concrete solutions for resident care will support improvements in this dimension in the coming years.

Finally, the baseline questionnaire highlighted two SC dimensions that were developing: Dimension 5 (*Compliance with procedures* (56.1%)) and Dimension 3 (*Teamwork* (60.9%)). Finally, Dimension 6 (*Staffing*) was underdeveloped (11.6%) [22]. Our analysis revealed that these scores did not change following the intervention. However, these themes were not specifically targeted by the project. Perceptions of the difficulty of complying with procedures are related to a high workload and low staffing levels. It is clear that French NHs must now consider recently-published actions and identify strategies to address these issues [27]. The Agency for Healthcare Research and Quality, for example, provides a list of targeted resources for each of the dimensions [28].

Drivers to improve SC scores

A previous publication [22] addressed the factors that explain the situation. Knowledge of the explanatory factors driving the change in scores is timely from the point of view of improving the system, and its large-scale deployment. Consequently, we studied the relationship between the evolution in SC scores and variables used to describe the sample of NHs (their structural profile, and the strategic choices made by top management in terms of quality and safety of care) on the one hand, and, on the other hand, conditions for the implementation of the support system.

Baseline results highlighted that a poorly-developed SC was linked to existing quality and/or RM initiatives, and the presence of specialized, qualified professionals [22]. In this context, it is reasonable to assume that being familiar with RM terminology contributed to a critical understanding of the situation.

However, the present analysis found that these two factors did not explain improvements in SC scores. One hypothesis for this finding is that NHs initially approached the RM process based on their understanding of standard tools used in hospital structures. However, one of the objectives of the project was to redefine the notion of an AEAC, by expanding it to include the idea of the living environment and resident support. At the same time, it was important that the solution was simple and viable for all NHs, including those that did not employ a qualified, specialized RM professional. These considerations may have helped to bring RM approaches up-to-date in the supported NHs. Another important issue was the existence of a Quality policy. This aspect emerged as a lever for the development of two dimensions (*Overall perceptions of resident safety - organizational learning,* and *Staffing*). It should be noted that the support system is based on tools, such as action plans and monitoring, that are usually used in Quality approaches.

The baseline analysis found that having a capacity of more than 80 residents was associated with a poorer perception of SC [22]. It is reasonable to assume that this is linked to a lack of fluidity in communication between different departments and their staff. However, the present analysis found that this was not an explanatory factor in the evolution of SC scores. Instead, it appears that the system emphasizes the importance of exchanges between professionals, particularly for the definition and communication of action plans.

The mean Dependency score emerged as a factor in the evolution of SC, in particular Dimension 6 (*Staffing*). It should be noted that in France, an increase in the dependency of

residents increases the financial aid paid to NHs, allowing the recruitment of more care professionals.

Finally, no relationship was found between change in SC scores and the legal status of the NH. It is therefore not possible to conclude that this variable constitutes either a brake or a lever to the implementation of the system. On the other hand, being hospital-based contributed to improved scores for Dimension 4 (*Supervisor expectations and actions promoting resident safety*). In France, managers can be shared between NHs and hospitals. In this case, NHs naturally benefit from a more mature attitude to patient safety.

No relationship was found between change in SC scores and the presence of professionals (including top management) at the awareness-raising session. The same was true for the stability of the steering group. On the other hand, greater leadership among the steering group was consistent with improved perceptions of SC, particularly with regard to Dimension 1 (*Overall perceptions of resident safety - organizational learning*) and Dimension 5 (*Compliance with procedures*). It was clear that the members of the steering group played a key role in facilitating and monitoring the implementation of the RM process. Together, an established Quality and RM approach, and a steering group that shows leadership appeared to be the key levers for the observed improvement in SC scores.

Strengths and limitations of the EHPAGE study

EHPAGE is the first study in France to measure the impact of a support system on the SC of NH professionals. This research shows that this multimodal RM intervention, which ran over

many months, was accepted and implemented by the majority of participants. The project was fully adopted by 90% of NHs, and the reasons for dropping out were independent of the scheme (notably due to issues linked to staff absences or changes in top management). The study also shows that the intervention can improve dimensions of SC among professionals working in NHs. Our results are all the more remarkable given that the study was conducted at the NH level (rather than the unit level usually adopted for interventions conducted in hospitals), in a small number (58) of NHs, with a short period of time (18 months) between the two SC measures. Running the study over a period of at least three to five years [16], and with a larger sample of NHs could confirm our results. It would also allow us to improve the robustness of the explanatory factors, and avoid certain measurement hazards (such as the percentage of steering group members who wanted to use the RM knowledge they had acquired, which was linked to a negative change in SC scores in the present study).

The questionnaire response rate (64% in 2016 and 56.5% in 2017), although comparable with other studies (66% [11], 69% [29]), may also constitute a selection bias. However, the high number of questionnaires that were administered, and the research team's knowledge of the field may have helped to mitigate this bias. We identified some differences between the characteristics of respondents between 2016 and 2017, and it would clearly be interesting to examine the characteristics of non-respondents and any changes in adherence to the project. However, the present data do not support an explanation of these differences, and it is reasonable to assume that, given the small numbers, any potential selection bias resulting from differences in respondent profiles would have an insignificant impact on the evolution of SC scores. Finally, the fact that participation was voluntary, and

that only two French *départements* were represented, means that the results cannot be extrapolated to the entire country.

The literature highlights the difficulty of comparing results between international studies measuring SC and/or describing interventions to improve patient safety. This is thought to be due to the preponderance of studies conducted in healthcare (versus NH) settings, and the heterogeneity of the specificities of NH operations between countries [30]. Another consideration is the design of the intervention, as each program has specific objectives [17,18,31], along with differences in the psychometric validation of questionnaires, which can result in different aggregations of dimensions [11,13,31,32].

The application of the EHPAGE project in the current health context

If the EHPAGE project was extended in participating NHs, it would make sense to repeat the measurement of SC. As Cappelen et al. [31] point out, few longitudinal studies have explored the durability and variability of SC. The EHPAGE system was designed to ensure that the intervention would be sustainable—notably, facilitators from the research team were in contact with members of the RM steering group, there was a positive commitment from top management. The literature notes that the presence of facilitators is a key to the success of interventions in both the short and the long term [33]. Their presence also encourages top management to create and support environments in which staff feel responsible for the SC of residents [31].

The EHPAGE intervention can be improved. Consideration could be given to the integration of residents' families or their representatives into the system. The failure to include these external actors in the process of improving healthcare safety and quality is all the more damaging as they have a different perspective, and often have an insight into more intimate aspects of residents' lives (their physical care, anxieties, feeling of institutionalization, relationships with professionals or other residents, etc.), which are essential to take into account. In addition, as the French population ages, with a concomitant increase in chronic disease, health systems will evolve towards more home and outpatient care, requiring increased coordination between all actors, including those who are cared for and those who provide care. Therefore, and following Storm et al. [34], further thought needs to be given to a collaborative analysis that crosses organizational boundaries between different actors and structures (hospital or in-home care) and examines how care can best be provided. Finally, the challenges associated with the quality of the working life of professionals who provide care should not be forgotten. The importance of allocating time for the team to reflect (known as Crew Resource Management Health) has never been truer than now.

Conclusion

In France, neither local nor national policies address the full range of SC dimensions in NHs. Faced with the massive challenge of an ageing population, and the associated changes to the care of residents in NHs, it seems mandatory to adopt a systematic and integrated approach to the safety of care [35].

In this context, QualiREL Santé developed a support system for NHs to implement their RM approach. This system, which is all the more important given the current COVID-19 pandemic, should ultimately improve the care of residents by developing a SC among NH professionals. Our findings show that the system was well-received, and implemented by participating NHs. Its impact was measured quantitively via the NFSOPS-F questionnaire. The analysis found that the improvement in Dimension 7 (*Feedback and communication about incidents*) was statistically significant, and that improvement in Dimension 1 (*Overall perceptions of resident safety - organizational learning*) was close to significant (+3.1%; *p*-value=0.044). Our findings highlighted that the system was all the more effective in improving SC if the NH had already initiated a Quality approach. They also emphasize that it is important for top management to work with professionals who show leadership in the implementation of the RM approach.

To conclude, it is now up to each participating NH to keep the process alive. The system, which is now fully digitized, is currently being adapted to Belgian hospitals by a structure that is similar to QualiREL Santé.

Acknowledgements

The authors would like to thank the DGOS for its help. The authors would like to thank all 61 NHs who took part in the EHPAGE project. The authors would like to thank Elaine Seery at *Traduction Edition Scientifique* for translation and editing services.

References

1. Amalberti R, Vincent C, Nicklin W, Braithwaite J. Coping with more people with more illness. Part 1: the nature of the challenge and the implications for safety and quality. Int J Qual Health Care. 2019; 3: 154–8.

2. Teigné D, Lucas M, Leclère B, Moret L, Terrien N. Panorama des domaines de risques associés aux soins en Ehpad [Overview of the risk areas associated with care in nursing homes]. Gériatrie Psychol Neuropsychiatr Viellissement. 2017; 15(2): 117–26.

3. Direction de la recherche, des études, de l'évaluation et des statistiques. Etudes et Résultats - 728000 résidents en établissements d'hébergement pour personnes âgées en 2015 [Directorate for Research, Studies, Evaluation and Statistics. Studies and results - 728,000 residents in residential institutions for the elderly in 2015]. 2017. https://drees.solidarites-sante.gouv.fr/sites/default/files/er1015.pdf. Accessed 18 Jun 2021.

4. Direction Générale de l'offre de soins, Direction Générale de la Santé, Haute Autorité de Santé. Programme national pour la sécurité des patients 2013–2017 [National Patient Safety Programme 2013–2017]. 2013. http://solidarites-sante.gouv.fr/IMG/pdf/programme_national_pour_la_securite_des_patients_2013-2017-2.pdf.

5. Wagner LM, Rust TB, Canadian Patient Safety Institute. Safety in long-term care settings: broadening the patient safety agenda to include long-term care services. Edmonton: CPSI; 2008. http://www.patientsafetyinstitute.ca/English/research/commissionedResearch/SafetyinLongTermCareSettings/Documents/Reports/LTC%20paper%20-%20Safety%20in%20LTC%20Settings%20-%202008.pdf. Accessed 19 Dec 2019.

6. La sécurité des patients. Mettre en œuvre la gestion des risques associés aux soins en établissement de santé. Des concepts à la pratique [Patient safety. Implementing risk management in health care institutions. From concepts to practice]. 2012. http://www.has-sante.fr/portail/upload/docs/application/pdf/2012-04/guide_gdr_pages1a64.pdf. Accessed 24 Oct 2016.

7. Guldenmund FW. The nature of safety culture: a review of theory and research. Saf Sci. 2000;34:215–57.

8. Kristensen S, Bartels P. Use of patient safety culture instruments and recommendations. Aarhus, Denmark, European Society for Quality in HealthCare - Office for Quality Indicators. 2010.

9. Shortell S, Gillies R, Anderson D, Erikson K, Mitchell J. Remaking health care in America. Hosp Health Netw. 1996; 6: 43–4.

10. Westat R, Sorra J, Franklin M, Streagle S. Survey User's Guide Nursing Home Survey on Patient Safety Culture. 2008. https://www.ahrq.gov/sites/default/files/publications/files/nhguide.pdf. Accessed 31 Mar 2017.

11. Zúñiga F, Schwappach D, De Geest S, Schwendimann R. Psychometric properties of the Swiss version of the Nursing Home Survey on Patient Safety Culture. Saf Sci. 2013;55:88–118.

12. Lin S-Y, Tseng WT, Hsu M-J, Chiang H-Y, Tseng H-C. A psychometric evaluation of the Chinese version of the nursing home survey on patient safety culture. J Clin Nurs. 2017; 26: 4664–74.

13. Thürmann P. Arzneimitteltherapiesicherheit in Alten und Pflegeheimen: Querschnittsanalyse und Machbarkeit eines multidisziplinären Ansatzes [Cross-sectional analysis and feasibility of a multidisciplinary approach]. 2009 p. 58. https://www.bundesgesundheitsministerium.de/fileadmin/Dateien/5_Publikationen/Gesundheit/Berichte/Abschlussbericht_Arzneimitteltherapiesicherheit_in_Alten_und_Pflegeheimen_Querschnittsanalyse_und_Machbarkeit_eines_multidisziplinaeren_Ansatzes.pdf. Accessed 18 Dec 2019.

14. Teigné D, Mabileau G, Anthoine E, Lucas M, Leclère B, Moret L, et al. Transcultural adaptation and psychometric study of the French version of the nursing home survey on patient safety culture questionnaire. BMC Health Serv Res. 2019; 19: 490.

15. JORF. Décret n° 2016-1606 du 25 novembre 2016 relatif à la déclaration des événements indésirables graves associés à des soins et aux structures régionales d'appui à la qualité des soins et à la sécurité des patients [Decree 2016-1606 of 25 November 2016 on the reporting of serious adverse events associated with healthcare and regional support structures for healthcare quality and patient safety]. 2016. https://www.legifrance.gouv.fr/eli/decret/2016/11/25/AFSP1624746D/jo/texte. Accessed 16 Jan 2020.

16. Halligan M, Zecevic A. Safety culture in healthcare: a review of concepts, dimensions, measures and progress. BMJ Qual Saf. 2011; 20: 338–43.

17. Marshall M, Pfeifer N, de Silva D, Wei L, Anderson J, Cruickshank L, et al. An evaluation of a safety improvement intervention in care homes in England: a participatory qualitative study. J R Soc Med. 2018; 111: 414–21.

18. Johannessen T, Ree E, Strømme T, Aase I, Bal R, Wiig S. Designing and pilot testing of a leadership intervention to improve quality and safety in nursing homes and home care (the SAFE-LEAD intervention). BMJ Open. June 2019;9(6):e027790.

19. Xie J, Ding S, Zhong Z, Zeng S, Qin C, Yi Q, et al. A safety culture training program enhanced the perceptions of patient safety culture of nurse managers. Nurse Educ Pract. 2017; 27: 128–33.

20. Banaszak-Holl J, Reichert H, Todd Greene M, Mody L, Wald HL, Crnich C, et al. Do Safety Culture Scores in Nursing Homes Depend on Job Role and Ownership? Results from a National Survey. J Am Geriatr Soc. 2017; 65: 2244–50.

21. Titlestad I, Haugstvedt A, Igland J, Graue M. Patient safety culture in nursing homes – a cross-sectional study among nurses and nursing aides caring for residents with diabetes. BMC Nurs. 2018; 17: 36.

22. Teigné D, Mabileau G, Moret L, Terrien N. What is the level of safety culture in French nursing homes? The EHPAGE study. BMC Health Serv Res. 2021; 21: 1332.

23. TeignÉ D, Mouret D, Lucas M, Gaultier A, Moret L, LeclÈre B, et al. Incidence des événements indésirables associés aux actes de soins et d'accompagnement en Ehpad : projet EHPAGE [Incidence of adverse events associated with technical care and support in French nursing homes: the EHPAGE project]. Gériatrie Psychol Neuropsychiatr Viellissement. 2019; 17(3): 243–53.

24. Anderson JC, Gerbing DW. The effect of sampling error on convergence, improper solutions, and goodness-of-fit indices for maximum likelihood confirmatory factor analysis. Psychometrika. 1984; 49: 155–73.

25. Cronbach LJ. Coefficient alpha and the internal structure of tests. psychometrika. 1951; 16: 297–334.

26. Streiner D, Geoffrey R. Health Measurement scale: a Practical Guide to their Development and Use. 3rd ed. Oxford: Oxford University Press. 2003.

27. Haute autorité de santé (HAS). Enquêtes sur la culture de sécurité - Comprendre et agir [Safety Culture Surveys - Understanding and acting]. 2019 https://www.has-sante.fr/upload/docs/application/pdf/2019-06/spa_123_guide_culture_securite_cd_2019_05_29_v1.pdf. Accessed 16 Jan 2020.

28. AHRQ (Agency for Healthcare Research and Quality). Improving Patient Safety in Nursing Homes: A Resource List. 2016; 27.

29. Cappelen K, Harris A, Aase K. Variability in staff perceptions of patient safety culture in Norwegian nursing homes—a longitudinal cross-sectional study. Saf Health. 2018; 4: 9.

30. Winsvold Prang I, Jelsness-Jørgensen L-P. Should I report? A qualitative study of barriers to incident reporting among nurses working in nursing homes. Geriatr Nur (Lond). 2014; 35: 441–7.

31. Cappelen K, Harris A, Aase K. Variability in staff perceptions of patient safety culture in Norwegian nursing homes—a longitudinal cross-sectional study. Saf Health. 2018; 4: 9.

32. Desmedt M, Petrovic M, Beuckelaere P, Vandijck D. Assessing Resident Safety Culture in Six Nursing Homes in Belgium: J Patient Saf. 2018; 1.

33. Hartmann CW, Mills WL, Pimentel CB, Palmer JA, Allen RS, Zhao S, et al. Impact of Intervention to Improve Nursing Home Resident–Staff Interactions and Engagement. The Gerontologist. 2018; 58: e291‐301.

34. Storm M, Schulz J, Aase K. Patient safety in transitional care of the elderly: effects of a quasi-experimental interorganisational educational intervention. BMJ Open. 2018; 8:e017852.

35. Braithwaite J, Vincent C, Nicklin W, Amalberti R. Coping with more people with more illness. Part 2: new generation of standards for enabling healthcare system transformation and sustainability. Int J Qual Health Care. 2019; 31: 159–63.

Supplementary Material

Table S1: Respondent characteristics for the 2016 and 2017 surveys.

Table S2: Indicators to assess the global fit of the final structural equation model on 2017 data (R output).

Table S3 : Details of univariate and multivariate models explaining the evolution of safety culture scores for the 7 dimensions according to NH parameters and the implementation of the system (n=28 NH)

Abbreviations

Adverse events associated with care (AEAC); Nursing Home (NH); Nursing Home Survey on Patient Safety Culture (NHSOPS); Risk Management (RM); Safety Culture (SC); Regional Support Structures (RSS).

Declarations

Ethics approval and consent to participate

The EHPAGE research protocol was approved by the Advisory Committee on Health Research Information Processing, and an ethics committee (*Groupe Nantais d'Ethique dans le Domaine de la Santé* [University Hospital of Nantes]). The participation of professionals in the research was the subject of prior information. Professionals were informed about the purpose of the research in advance. Participation was voluntary and informed consent was implied upon completion of the questionnaire (a procedure approved by the ethics

committee). According to articles L1121-1 and R1121-2 of the French Public Health Code, Institutional Review Board approval was not necessary.

Consent for publication

Not applicable.

Availability of data and material

The authors agree to share their data. Please contact the corresponding author should you require any of the results from this study.

Authors' contributions and guarantors' information

DT was the operative project leader, participated in the data collection, in the interpretation of the data and drafted the manuscript. GM performed the statistical analysis, interpreted the data, and helped to draft the manuscript. ML participated in the data collection and revised the manuscript. LM designed the study, and revised the manuscript. NT designed the study, was delegated investigator, participated in the interpretation of the data and revised the manuscript. All authors have read and approved the final manuscript.

DT, GM, and NT are contributors and guarantors for the overall content. The guarantors accept full responsibility for the work and/or the conduct of the study, had access to the data, and agreed to publish the results.

The corresponding author attests that all listed authors meet authorship criteria, and that no others meeting the criteria have been omitted.

Transparency declaration

The lead author affirms that this manuscript is an honest, accurate, and transparent account of the study being reported; that no important aspects of the study have been omitted; and that any discrepancies from the study as planned (and, if relevant, registered) have been explained.

Figure
Click here to access/download;Figure;Fig1.jpg

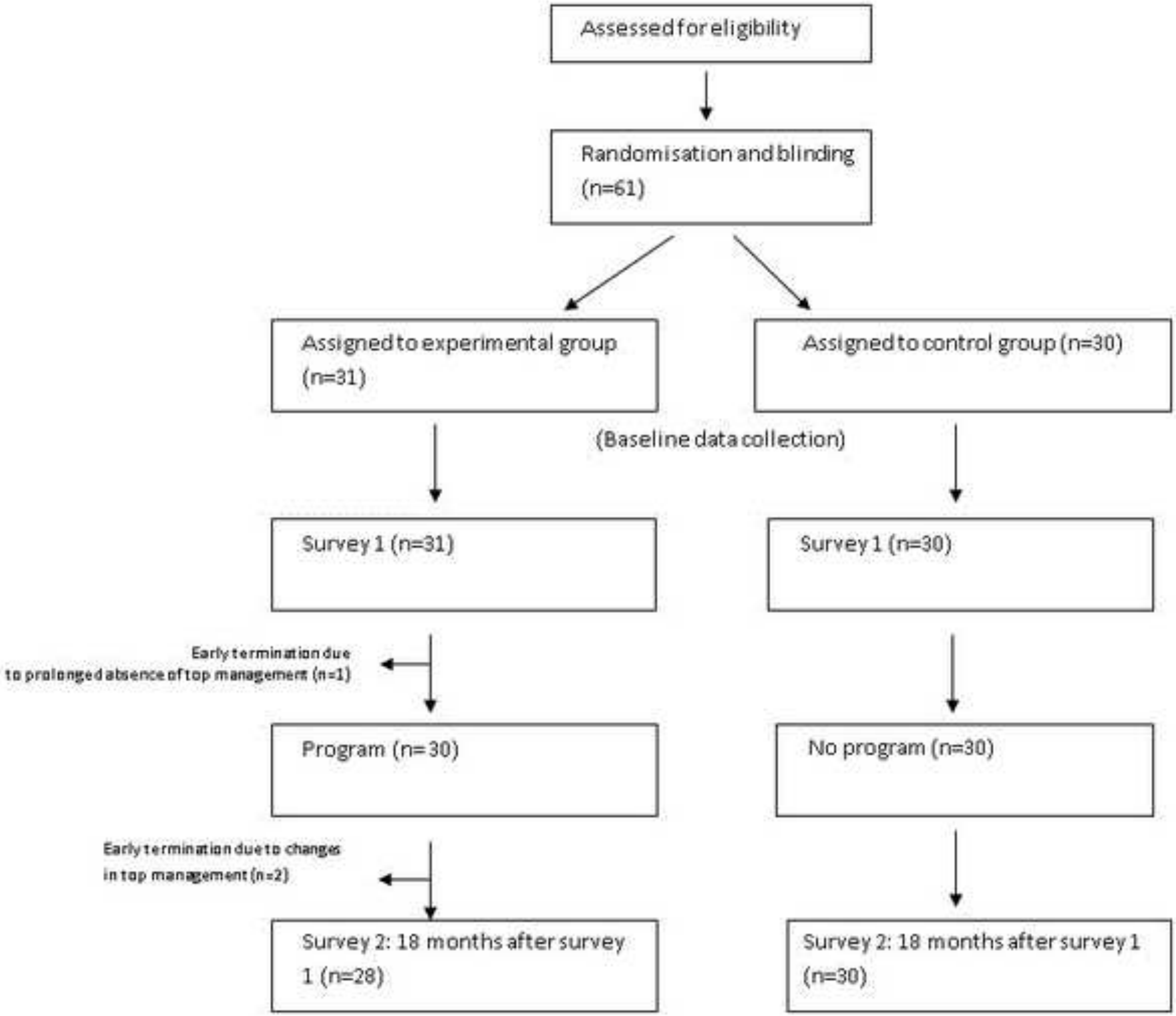

Click here to access/download
Supporting Information
TableS1Annex 1.doc

Click here to access/download
Supporting Information
TableS2Annex 2.doc

Click here to access/download
Supporting Information
TableS3Annex 3.docx
