# Supplementary material for: Safety culture in French nursing homes: A randomised controlled study to evaluate the effectiveness of a risk management intervention associated with care
Source: PLoS One. 2022 Dec 1;17(12):e0277121. doi: 10.1371/journal.pone.0277121 (PMC9714758; doi:10.1371/journal.pone.0277121)
Supplement: Attachment — Submitted filename: Safety culture reviewd articles.docx [file pone.0277121.s005.docx]

Dear editors, thank you for giving me a chance to review this manuscript, here are my comment.

**Reviewer Comments for a research entitled:** Safety culture in French nursing homes: a randomized controlled study to evaluate the 2 effectiveness of a risk management intervention associated with care.

1. In the abstract section line number 30-31 how voluntary participant be inclusion criteria and this may affect the principle of random allocation(justify it)
2. In the result of abstract section of the line number 41-43, <<After the intervention, the dimension Feedback and communication about incidents significantly improved (+2.8%; p-value=0.044), and so what was dimension of feedback and communication about safety practice or incident before an intervention?
3. In line number 53 to 66 in introduction section it is better to write about safety culture implementation in healthcare system and its importance in risk management rather than writing the characteristics of the population in that administrative region.
4. Is the tool is validated in your research context?
5. How determine the sample size for this study purpose?
6. Line number 124, randomization might be affected using voluntary participation of study participants as inclusion criteria rather than other exposure status and please explained it how was the selection process
7. Do you control researcher bias in assignments’ of in intervention in exposed group and unexposed group this is due to the researches should remain unaware of which treatment was given for which group until the study is completed?
8. Regarding to ethical concern of the research that involve human subject ethical concern is mandatory what types of consent you obtained from the study participants and before or after completion of the questioner participants was give their consent?
9. In line number 300-304, the result showed that presence of high non- response rate and did this affect your result?
10. In your discussion section it is better to start the discussion with the purpose of conducted this research followed by the main finding of your research.

In general the paper is very interesting and it’s very important to address problems of related with safety culture implementation in healthcare system (especially involvement of top management in SC promotion).

1. The article is needs rewrite in short.
2. The discussion part is shallow and it is better to add different literatures related with the concerned variables and it is so long but it needs short and precise to the concern.
3. Limitation and strength is so long it is better write in short .

Therefore, if this concern is address the paper will accepted for publication in your esteemed journal
